# Natural infection of *Didelphis aurita* (Mammalia: Marsupialia) with *Leishmania infantum* in Brazil

**DOI:** 10.1186/1756-3305-5-111

**Published:** 2012-06-07

**Authors:** João Carlos Araujo Carreira, Alba Valéria Machado da Silva, Daniela de Pita Pereira, Reginaldo Peçanha Brazil

**Affiliations:** 1 Núcleo Jacarepaguá, Instituto Oswaldo Cruz/Fiocruz, Rio de Janeiro, Brazil; 2 Laboratório de Bioquímica de Proteínas e Peptídeos, Instituto Oswaldo Cruz/Fiocruz, Rio de Janeiro, Brazil; 3 Laboratório de Bioquímica Molecular de Doenças Endêmicas, Instituto Oswaldo Cruz/Fiocruz, Rio de Janeiro, Brazil; 4 Laboratório de Bioquímica e Fisiologia de Insetos, Instituto Oswaldo Cruz/Fiocruz, Avenida Brasil, 4365, Rio de Janeiro, RJ, CEP 21045-900, Brazil

**Keywords:** *Didelphis aurita*, *Leishmania infantum*, Tissular parasitism, Amastigotes

## Abstract

**Background:**

The opossum *Didelphis* have been considered as natural hosts of *Leishmania* parasites in the New World, suggesting an important role in the epidemiology of Visceral Leishmaniasis (VL). Among six extant species that belong to the genus *Didelphis*, only two (*D. marsupialis* and *D. albiventris*), have been mentioned as natural hosts of *Leishmania infantum* in Brazil and Colombia. In the present paper, it is reported for the first time, the observation of intracellular parasites (amastigotes) in tissues of *Didelphis aurita* naturally infected with *Leishmania infantum* in Brazil. We also discuss some aspects associated to the relationship between *L. infantum* and the geographical distribution of some species of the genus *Didelphis*.

**Methods:**

The opossums studied were caught by wire traps (Tomahawk) in Barra de Guaratiba, a peri-urban area in Rio de Janeiro. The opossums were killed with an overdose of Thiopental sodium.At necropsy, macroscopic alterations were examined and samples from liver, spleen, lymph nodes, ear, abdominal skin, scent glands and bone marrow were collected for parasitological and molecular diagnoses.

**Results:**

Forty-eight opossums were captured in an AVL endemic region, 30 being caught in a mangrove area and eighteen animals in a forest area near to some residential-yards. Among the thirty opossums trapped in the mangrove area, all of them were negative by both imprint and sera samples assayed on Dipstick Tests, that is a test based on a combination of protein-A colloidal gold conjugate and rk39 *Leishmania* antigen to detect anti-*Leishmania* antibody in serum or plasma. At the macroscopic examination one out of eighteen opossums, caught close to the forest, presented alterations compatible with spleen hypertrophy and three were positive by Dipstick Tests (16.6%) and presented amastigotes in the spleen and in one of them, the parasites were also observed in a submandibular lymph node. *Leishmania infantum* infections were confirmed through dot blot hybridization using a *L. infantum*-specific biotinylated probe*.*

**Conclusions:**

In the present paper we present the first report of amastigotes in the tissues of *Didelphis aurita* (Mammalia: Marsupialia) naturally infected with *Leishmania infantum*. We also attempt to claim the particular role of some opossum species as hosts of *Leishmania infantum*, contributing at least in part on the description of potential sylvatic reservoirs.

## Background

Domestic and sylvatic canids are considered the most important reservoirs of *Leishmania infantum* in America [1,2]. Nevertheless, under certain conditions the opossums of the genus *Didelphis* could play an important part in the maintainance of the parasite, *Leishmania infantum,* due to its ability to live in different habitats providing a link between the wild and domestic cycles of the disease [3].

Likewise in urban endemic areas of leishmaniasis, where the majority of sylvatic mammals are not normally found, the opossums may also assume a very important role in the epidemiology of the disease.

The species *Didelphis albiventris* was the first non-canid wild mammal to be described as naturally infected with *Leishmania infantum* in the New World [4]. In this same study, it was also reported that *Lutzomyia longipalpis,* the main vector of the parasite in the New World, was capable of feeding on this species. At a later date, *L. infantum* was isolated from two *Didelphis albiventris,* which had been caught by trapping in a focus of American Visceral Leishmaniasis (AVL) in Bahia, Brazil [5].

Corredor *et al*. (1989) [6], working in one focus of AVL in Colombia observed that the infection-rate of the opossums (*D. marsupialis*) was as high as 12/37 (32.4%), concluding that this animal is an important reservoir of *L. infantum*. Additionally, Travi *et al*. (1998a) [7] studying the natural infection of *D. marsupialis* in both undisturbed and degraded dry forest in northern Colombia, observed respectively that 3/21 (14.3%) and 13/137 (9.5%) of those animals were infected. Cabrera *et al*. (2003) [3], studying an opossum population in Rio de Janeiro, Brazil, observed that 29% were positive with *L. infantum by* IFA, and the authors considered that the presence of opossums represented an important risk factor for the transmission of leishmaniasis.

Finally, Adler *et al*. (2003) [8], examining the feeding success of *Lutzomyia evansi,* who experimentally exposed small mammal hosts in an endemic focus in northern Colombia, concluded that *Didelphis marsupialis* is one of the species of wild mammals with the greatest impact on transmission of *L. infantum*.

Despite the considered importance of the opossums as reservoirs of *Leishmania infantum* as reflected by: high percentage of natural infections, species predominance, high adaptability to peridomiciliar habitats, vector attractiveness, concomitancy with human cases as well as the capacity to act as a link between sylvatic and domestic environment [3], there is only one record related to the direct observation of parasites in the tissues of *D. marsupialis* from Colombia with natural infection [6].

In addition, it is very important to understand the specific role of each species of the genus *Didelphis* on AVL epidemiology, considering that they present a wide geographical distribution on the Americas coincident with several countries where AVL is endemic. Until now, only two species of *Didelphis* have been studied.

In the present paper, we report for the first time, the observation of amastigotes in tissues of *Didelphis aurita* naturally infected with *L. infantum* trapped in an urban area of Rio de Janeiro state, Brazil. We also discuss some aspects related to the relationship between *L. infantum* and the geographical distribution of some species of the genus *Didelphis.*

## Methods

### Study area

The opossums studied were caught in Barra de Guaratiba, Rio de Janeiro a peri-urban endemic area for AVL. Several notified human cases have been reported from this area with a high animal host seroprevalence in dogs, cats and opossum [3,9] Barra de Guaratiba (23^°^ 03’ 57” S; 43^°^ 34’ 00” W) is a strip of land localized between the Atlantic Ocean and the mountains of Pedra Branca, a massif belonging to Serra do Mar in Rio de Janeiro State. The climate is tropical, with an annual average temperature of 27.5 °C and rainfall of 1400 mm. Secondary Atlantic Forest altered by anthropic action covers the mountain slopes.

### Opossum populations

Forty-eight opossums were captured by wire traps (Tomahawk), thirty were caught in a mangrove area and the rest of them in a forest area nearby some residential-yards. Blood was collected by puncture of the femoral artery, allowed to clot and serum separated by centrifugation and stored at −20 °C until use. The opossums were killed with an overdose of Thiopental sodium (10–25 mg/kg, i.v.) and potassium chloride (100 mg/kg, IV). All the animals used in this study were identified in accordance to Cerqueira (2007) [10].

At necropsy, macroscopic alterations were examined and samples from liver, spleen, lymph nodes, ear, abdominal skin, scent glands and bone marrow were collected for parasitological and molecular diagnoses. For the imprints, three samples of each tissues were fixed with methanol and stained with May Grünwald-Giemsa (SIGMA®). The dilution was two drops of Giemsa stain for each ml of distilled water and a staining time of 40 minutes was used.

All animals used in this study were in accordance with guidelines defined by the Committee of Ethics in Animal Experimentation of the Oswaldo Cruz Foundation Rio de Janeiro, Brazil and permission for trapping was granted by a license of Instituto Brasileiro do Meio Ambiente e dos Recursos Naturais Renováveis (IBAMA).

### Dipstick test

The Dipstick Test (kit Rapidest Leishmania®) was carried out from blood samples, according to the manufacturer’s instructions. The dipsticks were briefly placed into 10 μl of serum. After 10 min a red control line and, if positive, a second line appeared on the test field. The test is based on a combination of protein-A colloidal gold conjugate and rk39 *Leishmania* antigen to detect anti-*Leishmania* antibody in serum or plasma.

### PCR and dot blot analysis

For DNA isolation and PCR analysis, opossums’ spleen and liver samples, from positive Dipstick tested animals, were mixed with a solution of Guanidine-EDTA in a proportion of 1:1 and after 24 hours the mixture was boiled for 1 minute and kept at 4 °C until DNA extraction. Total DNA obtained from cultured *L. (L.) infantum* promastigotes (MHOM/BR/1974/PP75) were used as a positive control.

For the PCR multiplex assay, one primer pair was used. The primers [5′-GGCCCACTATATTACACCAACCCC-3′and 5′-GGGGTAGGGGCGTTCTGCGAA-3′] were employed to amplify the conserved region of kinetoplastic minicircle DNA from *Leishmania* spp [11]. The reactions were carried out in a final volume of 100 μl containing 1 × Taq polymerase buffer (Applied Biosystems, Foster City, CA, USA), 4.5 mM MgCl2, 200 μM of each dNTP (Invitrogen), 0.2 μM of each primer, 1.25 U Taq Gold DNA polymerase (Applied Biosystems) and 10μL DNA sample. The mixture was incubated in a Perkin-Elmer thermocycler (GeneAmp PCR System 9600; Applied Biosystems, Foster City, CA, USA). Ten microlitres of the amplification reaction product was resolved in 2% agarose gel electrophoresis and visualised under UV transillumination. The amplified products were also analysed by dot blot hybridization using a *L. infantum*-specific biotinylated probe [5`AAAAATGGGTGCAGAAAT 3`] [12]. Hybridization reactions were revealed using Luminol reagent (Santa Cruz Biotechnology). Each experiment included both negative and positive controls.

## Results

### Necropsy and dipstick tests

**Figure 1:**
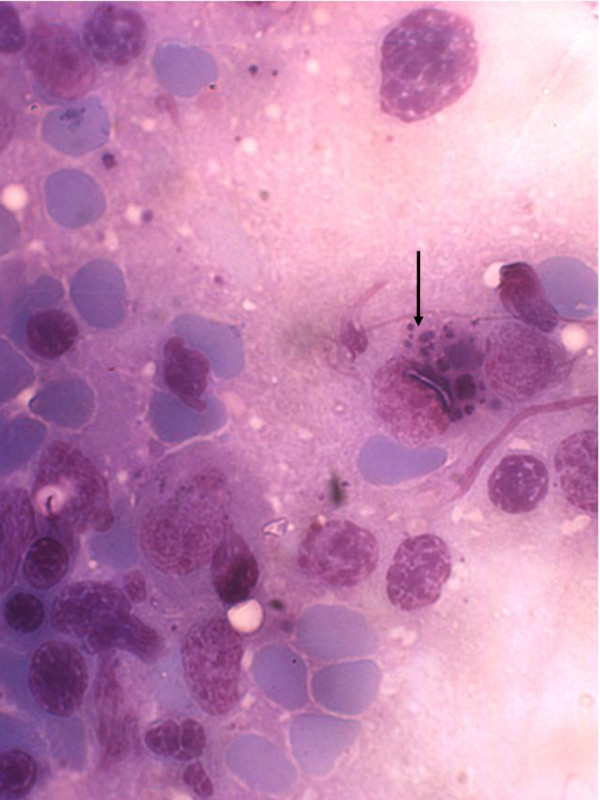
**Spleen imprint of*Didelphis aurita*(animal 1) from Barra de Guaratiba endemic area.** The Giemsa-stained imprint showing several *Leishmania infantum* amastigotes in the cytoplasm of a macrophage (arrow). (Original magnification, x1000).

Considering the analysis of both the imprint and sera samples assayed on Dipstick Tests, among the thirty animals trapped in the mangrove area, all of them were negative. Otherwise, it was observed that three out of 18 opossums caught close to the forest were positive (16.6%). At macroscopic examination one out of eighteen animals presented alterations compatible with spleen hypertrophy. All three positive animals presented amastigotes in spleen and in one of them the parasites were also observed in a submandibular lymph node. In the spleen (Figure 1) as well as in the lymph nodes, the parasites were observed in general inside macrophages with the cells showing the parasitophorous vacuoles containing easily detectable amastigotes (Table 1).

**Table 1:** Results of the three positive opossums by dipstick tests, imprints, PCR and macroscopic findings on the necropsy

**Animal**	**Dipstick**	**Imprint**		**PCR**	**Necropsy**
		Spleen	lymph node		
**1**	+	+	-	+	-
**2**	+	+	+	+	spleen hypertrophy
**3**	+	+	-	+	-

### PCR and dot blot

**Figure 2:**
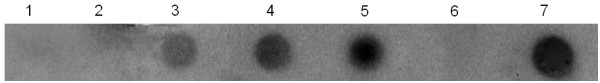
**Hybridization with specific probe for*Leishmania infantum.*** Amplified products from specific primers for the *Leishmania* (120 pb). (1) negative control, (3), (4) and (5) opossums positive samples, (2) and (6) opossums negative samples, (7) positive control. All samples studied were in duplicate.

For PCR and Dot Blot sample studies, in the visualization of the amplification products in agarose gel of the three opossums the samples were positive, amplifying a product of 120pb, corresponding to the conserved kDNA region of *Leishmania* sp*.* After hybridization of this amplified product with a specific probe for *L. infantum*, the positive samples were confirmed as *Leishmania infantum* and controls maintained negative (Figure 2).

## Discussion

In early studies, the role of *Didelphis albiventris* as a reservoir of *L. infantum* was unclear due to low rate of natural infections and discreet histopathological findings [4,13] . Subsequently, works with *Didelphis marsupialis* showed that it can exhibit a different infection pattern, with significant tissue parasitism and tissue lesions as well as high percentages of natural infections [3,5,13-15].

In our results, *D. aurita* showed a very similar infection profile as compared to *D. marsupialis*, so it is likely that black-eared opossums represented by those species could exhibit a higher susceptibility to *L.infantum* infections differently to the white-eared opossum, *D. albiventris*. This hypothesis is based on the fact that the genetic background at the inter-species level could influence the susceptibility to a determined parasite, as already observed in murine models for *Leishmania*[7,16,17].

Taking into account the several records related to natural infections of opossums with *L.infantum* in the New World, if we consider the six extant species of the genus *Didelphis* that are: *D.virginiana, D.pernigra, D.imperfecta, D.aurita, D.albiventris and D.marsupialis*[10,18,19], apparently only the last two have been described as natural hosts of the parasite, indicating a need for studies that evaluate the role of other species in the epidemiology of AVL [5,13-15,20] . Nevertheless, a very important point to be considered is the need of a correct classification of the species investigated, because the host’s behavior and its interplay with the habitat are key factors that determine the epidemiological characteristics of the AVL in a specific place.

Considering specifically the descriptions about the participation of didelphids in the epidemiology of leishmaniasis, we could mention some taxonomic misinterpretations where the authors described *D. marsupialis* as a host of *Leishmania*, respectively in Rio de Janeiro and the metropolitan region of Belo Horizonte, Minas Gerais, Brazil [3,21] . In fact, it is very unlikely that any specimens of *D. marsupialis* have been captured in these areas, as, based on the last reviews of the genus *Didelphis*[10,18,19]. this species is more often related to the Amazon region, thus, it is probable that they worked with *D. aurita* which occurs in southeastern Brazil and it is morphologically very similar to *D. marsupialis*.

## Conclusions

The high complexity of the AVL eco-epidemiology related to the geographical distribution of the several species that compound the genus *Didelphis* in South America have resulted in some mistakes, thus in the present paper we attempted to argue the probable role of some species of opossums correlated to their natural habitats, contributing at least in part to the elucidation of AVL ecology in relation to this group of marsupial hosts.

## Competing interests

The authors declare that they have no competing interests.

## Authors’ contributions

JCAC conceived of the study, besides acquisition, analysis and interpretation of data; drafting the article or revising it critically for important intellectual content. AVMS participated in the design of the study, analysis and interpretation of data. DPP carried out the molecular genetic studies. RPB helped to draft the manuscript and revised it critically for important intellectual content. All authors read and approved the final manuscript.
